# Molecular insights into STAT1a protein in rohu (*Labeo rohita*): unveiling expression profiles, SRC homology domain recognition, and protein-protein interactions triggered by poly I: C

**DOI:** 10.3389/fimmu.2024.1398955

**Published:** 2024-06-27

**Authors:** Basanta Kumar Das, Soumya Prasad Panda, Smruti Priyambada Pradhan, Subhashree Subhasmita Raut, Mala Kumari, Dharmendra Kumar Meena

**Affiliations:** ^1^ Aquatic Environmental Biotechnology (AEB) Division, Indian Council of Agricultural Research (ICAR) - Central Inland Fisheries Research Institute, Kolkata, West Bengal, India; ^2^ Riverine & Estuaries Fisheries Division, Indian Council of Agricultural Research (ICAR) -Central Inland Fisheries Research Institute, Kolkata, West Bengal, India; ^3^ Open Water Aquaculture Production and Management (OWAPM) Division, Indian Council of Agricultural Research (ICAR)-Central Inland Fisheries Research Institute, Kolkata, West Bengal, India

**Keywords:** STAT1a, innate immunity, interferon pathway, *Labeo rohita*, immune response, phylogenetic analysis, 3D modeling, molecular dynamics simulations

## Abstract

**Introduction:**

STAT1a is an essential signal transduction protein involved in the interferon pathway, playing a vital role in IFN-alpha/beta and gamma signaling. Limited information is available about the STAT protein in fish, particularly in Indian major carps (IMC). This study aimed to identify and characterize the STAT1a protein in *Labeo rohita* (LrSTAT1a).

**Methods:**

The full-length CDS of LrSTAT1a transcript was identified and sequenced. Phylogenetic analyses were performed based on the nucleotide sequences. The in-vivo immune stimulant poly I: C was used to treat various tissues, and the expression of LrSTAT1a was determined using quantitative real-time polymerase chain reaction (qRT-PCR). A 3D model of the STAT1a protein was generated using close structure homologs available in the database and checked using molecular dynamics (MD) simulations.

**Results:**

The full-length CDS of *Labeo rohita STAT1a* (*LrSTAT1a*) transcript consisted of 3238 bp that encoded a polypeptide of 721 amino acids sequence was identified. Phylogenetic analyses were performed based on the nucleotide sequences. Based on our findings, other vertebrates share a high degree of conservation with *STAT1a*. Additionally, we report that the *in vivo* immune stimulant poly I: C treatment of various tissues resulted in the expression of *LrSTAT1a* as determined by quantitative real-time polymerase chain reaction (qRT-PCR). In the current investigation, treatment with poly I: C dramatically increased the expression of *LrSTAT1a* in nearly every organ and tissue, with the brain, muscle, kidney, and intestine showing the highest levels of expression compared to the control. We made a 3D model of the STAT1a protein by using close structure homologs that were already available in the database. The model was then checked using molecular dynamics (MD) simulations. Consistent with previous research, the MD study highlighted the significance of the STAT1a protein, which is responsible for Src homology 2 (SH2) recognition. An important H-bonding that successfully retains SH2 inside the STAT1a binding cavity was determined to be formed by the conserved residues SER107, GLN530, SER583, LYS584, MET103, and ALA106.

**Discussion:**

This study provides molecular insights into the STAT1a protein in Rohu (*Labeo rohita*) and highlights the potential role of STAT1a in the innate immune response in fish. The high degree of conservation of STAT1a among other vertebrates suggests its crucial role in the immune response. The in-vivo immune stimulation results indicate that STAT1a is involved in the immune response in various tissues, with the brain, muscle, kidney, and intestine being the most responsive. The 3D model and MD study provide further evidence of the significance of STAT1a in the immune response, specifically in SH2 recognition. Further research is necessary to understand the specific mechanisms involved in the IFN pathway and the role of STAT1a in the immune response of IMC.

## 1 Introduction

Signal Transducers and Activators of Transcription (STAT), including mammalian STAT1, STAT2, STAT3, STAT4, STAT5a, STAT5b, and STAT6 (1), are a wide family of cytoplasmic proteins that take a role in signal transduction (2). They mediate cellular transcriptional responses during cytokine production, embryogenesis, and proliferation (3–5). STATs have conservative domains: amino-terminal, coiled-coil, Src homology 2 (SH2), linker, DNA binding, and transcriptional activation (6). When Janus kinase (JAKs) phosphorylate STATs, the receptor separates, forming active dimers or tetramers that move into the nucleus and attach to particular DNA regions to trigger the activation of target genes (7). The significance of *STAT1* in the interferon (IFN) response and innate immunity in teleost fish and mammals is widely recognized (8, 9). Research conducted on a neuroblastoma cell line revealed that *STAT1*-dependent inhibition of viral infection and replication occurred (10). Moreover, *STAT1*-deficient mice become susceptible to infection from primary dengue virus and pulmonary mycobacterium (11, 12). In human cell lines lacking *STAT1*, interferon-signalling is saved by zebrafish *STAT1* (4). Another gene analysis result suggested that *STAT1* could be activated in *Carassius auratus L.* blastulae embryonic (CAB) cells upon stimulation with poly I: C (13). It has been observed that the host antiviral response signals through STAT1 protein and fish IFN increase the production of ISGs in crucian carp (14). Various STAT1 proteins have been cloned and utilized to examine the immunological response in salmon, mandarin fish, and olive flounder (15–17). These findings imply that fish STAT1 shares many of the same functional characteristics and protein structure as human STAT1 and that fish *STAT1* is crucial to the fish’s innate immune system and antiviral defences.

The mechanism underlying IFN-induced signalling in fish is yet unknown due to the complexity and diversity of fish IFN systems. Fish have been found to possess two type II IFN genes, IFN-γ and IFN-γrel, in contrast to mammals (18). What’s more, the important molecules involved in the IFN-driven signalling pathway, such as Janus kinase (JAKs) and signal transducers and activators of transcription (*STATs*), contain two or more multi-copies in fish (19). For example, *STAT1a* an d *STAT1b* have been cloned from black carp (20). Two copies of *STAT1a* and *STAT1b* make up the STAT1 in zebrafish (21). According to Sobhkhez et al. (2014), three STAT2 homologs *STAT2a, STAT2b, and STAT2c* were found in Atlantic salmon. By elucidating the functions of fish *STATs*, we can get fresh perspectives on *STAT* function and a thorough understanding of fish signal transduction. The present study report on the cloning and characterization of the *STAT1a* gene from *L. rohita.* The main objectives of this study were (1) The first phase involved cloning the *LrSTAT1a* gene and analysing its expression using immunostimulants (Poly I: C) and (2) The second experiment was an *in-silico* investigation of the predicted protein of *LrSTAT1a* transcript. According to this study, the antiviral response in *L. rohita* might be caused by poly I: C activating STAT1a gene expression.

## 2 Materials and methods

### 2.1 Declaration of ethics

The Central Inland Fisheries Research Institute (ICAR) ethical committee at Barrackpore, Kolkata 700120, West Bengal, India, authorized this work. The Committee on Animal Ethics approved this experiment. (Approval date: 10/09/2019; approval code: CIFRI/EC/2019/62) The Institute’s defined protocols were followed when handling the fish used in the experiment.

### 2.2 Fish

Healthy *Labeo rohita* (weight 70 ± 10g) were obtained from the ICAR-Central Inland Fisheries Research Institute hatchery in Barrackpore, Kolkata 700120, West Bengal, India, for the *in-vivo* gene expression experiments. The fish were kept in a 500-liter tank. Throughout the experiment, the water temperature ranged from 25 to 30 degrees Celsius, and its pH was 7.5–7.8 with continuous aeration and a 20% water change every alternate day. The fish were given commercial floating feed twice a day and allowed to acclimate for two weeks before the start of the experiment.

### 2.3 Experiments design

The entire experiment was conducted in two steps, one after the other. (1) The first phase involved cloning the *LrSTAT1a* gene and analysing its expression using immunostimulants (Poly I: C – a synthetic analogue of ds RNA, Sigma, St. Louis, MO, USA). (2) The second experiment was an *in-silico* investigation of the predicted protein of *LrSTAT1a* transcript.

### 2.4 Immunostimulants and sample collection

Twenty- one fish was given intraperitoneal (IP) injections of 0.1 mL of poly I: C (Sigma, St. Louis, MO, USA) (1 mg/mL) PBS solution as stimulants, while another set of fish was kept as a control (Each day we collected the tissue samples from 6 nos. of fish where three nos. fish treated and 3nos. fish from control). All of the fish were grown in separate water tanks with appropriate care. The experiment was run for 14 days. Following fish anaesthesia and sacrifice, 20–50 mg of various tissues, including the brain, spleen, liver, intestine, heart, kidney, gills, and muscle, were separately collected at various time points (days 1, 2, 3, 4, 5, 7, and 14), and they were immediately stored in RNAlater (Amibion, USA) at -80°C for the *LrSTAT1a* expression study.

### 2.5 cDNA cloning and semi-quantitative PCR expression of LrSTAT1a

Total RNA was extracted from *L. rohita* samples using Trizol (Invitrogen, USA). cDNA template was synthesized from the total RNA using GoScript™ Reverse Transcription System (Promega, USA). The full-length cDNA of *LrSTAT1a* was obtained via RACE-PCR (rapid amplification of cDNA ends) using the SMARTer™ RACE cDNA Amplification Kit (Clonetech, USA). Specific primers (**Table 1**) were designed according to the putative coding sequences obtained from the NCBI database. To the core cDNA fragments of *LrSTAT1a* primers, *LrSTAT1a*FP1 forward and *LrSTAT1a*RP1 reverse were used for the primary PCR. The PCR amplification was done under the following conditions: initial denaturation at 95°C for 5min followed by 40 cycles of 95°C/45sec, 56°C for 30sec and 72°C for 45sec and final extension at 72°C for 7min. To clone the 5′ fragments, nested PCR was performed using four sets of specific primers (*LrSTAT1a*FP2, *LrSTAT1a*RP2; *LrSTATa1*FP3, *LrSTAT1a*RP3; *LrSTAT1a*FP4, *LrSTAT1a*RP4; *LrSTAT1a*FP5, *LrSTAT1a*RP5). Similarly, primer pairs were used to produce the t3′ fragments. The PCR segments were sequenced by Barcode Bioscience Pvt. Ltd. (Bangalore, India) after being ligated to the pMD19-T vector.

**Table 1:** Primers used in this study.

Primer name	Gene	Sequences (5’-3’)	Fragment (bp)	Tm (°C)	Applications
*LrSTAT1a*-FP1	*STAT1a*	5’-GTCGACATTTTACCATGATGG-3’	631	59.6	cDNA cloning
*LrSTAT1a*-RP1		5’-GCACATCCTCCAGAACCTGAATA-3’			
*LrSTAT1a*-FP2	*STAT1a*	5’-GAGTATCACGATGGAACAGC-3’	797	58.0	cDNA cloning
*LrSTAT1a*-RP2		5’-CACTGGATTCCTCTAAGTTC-3’			
*LrSTAT1a*-FP3	*STAT1a*	5’-GTCTCTATTGACAAAGATTTAACAG-3’	755	59.0	cDNA cloning
*LrSTAT1a*-RP3		5’-GTGATTCCTCCGTCTCTACAG-3’			
*LrSTAT1a*-FP4	*STAT1a*	5’-CACCTGCTCAACATCTGGAATGAT-3’	817	63.6	cDNA cloning
*LrSTAT1a*-RP4		5’-CATGCAACATGCAAGTTTCTTTATC-3’			
*LrSTAT1a*-FP5	*STAT1a*	5’-CACTTGTGTGAAGAAGAGGAGCCA-3’	705	67.7	cDNA cloning
*LrSTAT1a*-RP5		5’-TTCAATACAGTCATTTCATTTA-3’			
*LrSTAT1a*-73	*STAT1a*	5’-TCCAGGAGGATCCTGTTCAC-3’	146	58.4	RT-PCR
*LrSTAT1a*-73		5’-GGCCAGCTCATTTTGTTGTT-3’			
β – actin F	*β– actin*	5’-TTCGAGCAGGAGATGGGCACTG-3’	254	55	β - actin
β – actin R		5’-GCATCCTGTCAGCAATGCCA-3’			

### 2.6 QPCR analysis for the expression of *LrSTAT1a* mRNA

According to Das et al. (2019) and Panda et al. (2023), the *LrSTAT1a* gene expression analysis using qRT-PCR was conducted using the Light Cycler^®^480 detection system (Roche, Germany). A total of 0.5µl of 25 nanomolar for each forward and reverse primer, 2x asymmetrical cyanine dye, 5µl of SYBR Green, 1µl of master mixture (Roche Germany), 1mg/µl of cDNA and 3µl of nuclease-free water was used to make the qPCR reaction mixture. To amplify the 146 bp *LrSTAT1a* fragment, PCR reactions were set up with triplicate samples and 40 cycles of 95°C/10 sec, 55°C/10 sec, and 72°C/10 sec. Primary denaturation was then carried out for 10 min at 95°C. Using β-actin as a standard gene, the 2^-ΔΔCT^ method was used to determine the relative expression of the *LrSTAT1a* gene. Following PCR, the products were run on one-point-five percent (1.5%) agarose gel electrophoresis to verify the desired amplified product’s quality and gene expression. The housekeeping gene β-actin was used in this method to normalize fold change compared to the control gene. The average value added along with the estimated standard error was how the qRT-PCR data were expressed. The efficiency of the ladder (E) and the Ct deviation of the analysed amplicons with the corresponding reference gene were used to determine the relative expression (R) of the *LrSTAT1a* transcript.

A qRT-PCR was carried out to determine the relative expression level of *LrSTAT1a* in various tissues after Poly I: C induction. Fish injected with PBS were used as the negative control, whereas fish treated with Poly I: C was used as the positive control. The brain and kidney were chosen because they are considered the target organs for research on viral contagion.

### 2.7 Finding the significance

The statistical analysis tool SPSS was used to examine all the data (version 16.0 SPSS, USA). The differences in significance between the various treatment groups were compared at the P≤ 0.05 percent significant level using a one-way ANOVA followed by Duncan’s multiple range tests.

### 2.8 Amplification of the full-length *LrSTAT1a* gene by RACE-PCR

According to Das et al. (2019), RACE was performed using the GeneRacer ™ method (Invitrogen, USA) to retrieve the complete *LrSTAT1a* cDNA sequence. RACE involves RNA ligation, mRNA capping to modify the 5´ end of the mRNA, and precise RNA oligo selection using the T4 RNA ligase enzyme. CIP was combined with total RNA to remove 5’-phosphates. Additional truncation of mRNA and non-mRNA occurred without altering the genetic composition of capped and full-length mRNA. TAP was applied to capped (dephosphorylated) RNA to remove the 5’cap configuration from the mRNA. Using GeneRacer™ RNA oligo, this procedure guaranteed the existence of 5’-phosphate, which is a necessary precursor for ligation. The 26-base oligo GeneRacer™ 3′ primer had the sequence 5′-GCTGTCAACGATACGCTACGTAA CG-3′ with a Tm of 78°C. A 26-base oligo with the sequence 5′-GGACACTGACATGGACTGAAGGA GTAGTA-3′ with a Tm of 78°C was the GeneRacer™ 5′ nested primer.

There were multiple phases involved in the RACE procedure using a kit from Invitrogen, USA. The phenol-chloroform technique was used to precipitate and purify the RNA before enzymatic treatment. The RNA was decapped on the first day, and the ligated RNA was stored in ethanol for precipitation overnight. The PCR product was cloned using TOPO^®^ on the second day, followed by reverse transcriptase PCR and sequencing of plasmid DNA containing either the 3′ or 5′ PCR product.

Primers for the 5’ and 3’ RACE PCR, however, were created for the corresponding end sequences of the first cloned 360 bp (*LrSTAT1a*-FP1 and *LrSTAT1a*-RP1) and 146 bp cDNA fragments prior to performing RACE PCR (**Table 1**) methods.

### 2.9 *STAT1a* transcript *in-silico* analysis and phylogenetic tree construction

**Figure 1:**
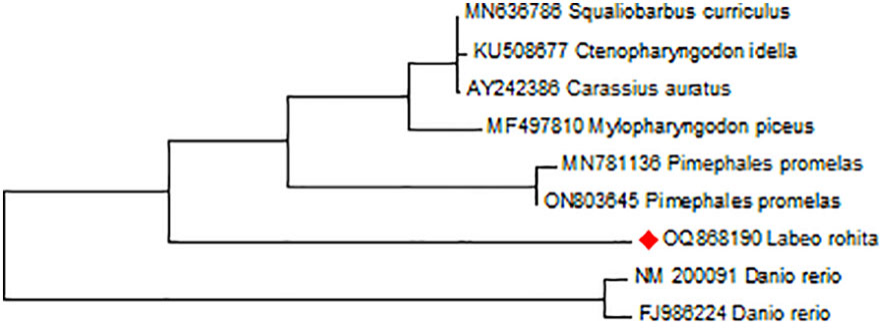
Showing the evolutionary homology of *L. rohita STAT1a* nucleotide sequence produces by MEGA 11 software. Branches were validated by cluster in the taxa in 1000 replicates of bootstrap that are represented as a percentage shown in each branch node. The phylogenetic tree was analysed using the neighbour-joining criteria for branching.

After the full-length *LrSTAT1a*, CDS were generated from the sequence contigs and analysed through various bioinformatics analyses. *LrSTAT1a* amino acid sequences were inferred by using the ExPASy website (https://web.expasy.org/translate/). The *L. rohita STAT1a* gene’s open reading frames (ORFs) were predicted using the NCBI ORF finder, and the longest ORF was selected for further investigation. Using the ProtParam tool of ExPaSy, the physicochemical characteristics of the encoded STAT1a protein were ascertained. The molecular weight (MW) and isoelectric point (pi) of STAT1a protein were calculated by Expasy tool. The grand average of hydropathicity (GRAVY) was calculated by ProtParam of Expasy tool with protein sequence of *STAT1a* gene. The motifs of STAT1a protein were identified by motif tool of Genome Net. The promoter region of *STAT1a* gene was extracted from the NCBI database by taking 1000 bp of genomic DNA sequence upstream to the *STAT1a* gene. Several TATA boxes were identified by OProf of Expasy tool in predicted promoter region of *STAT1a* gene. Using the MEGA version 11 tool and the multiple sequence alignment of the STAT1a proteins, the neighbour-joining (NJ) tree was inferred (**Table 2**; **Figure 1**). The bootstrap test with 1000 repeats was used to assess the reliability of the phylogenetic tree.

**Table 2:** Lists the STAT1 family members' accession numbers that were used to build the phylogenetic tree for this investigation.

Gene	Species	Accession No.
*STAT1a*	*Labeo rohita*	OQ868190
*STAT1*	*Squaliobarbus curriculus*	MN636786
*STAT1*	*Carassius auratus*	AY242386
*STAT1*	*Ctenopharyngodon idella*	KU508677
*STAT1*	*Mylopharyngodon piceus*	MF497810
*STAT1*	*Pimephales promelas*	MN781136
*STAT1*	*Pimephales promelas*	ON803645
*STAT1*	*Danio rerio*	NM_200091

### 2.10 Protein and ligand preparation

We used the Robetta web server (https://robetta.bakerlab.org/) to generate the initial model structure using RoseTTA Fold as the algorithm.

PROCHECK (https://saves.mbi.ucla.edu/) was used to validate the protein models. It helps create the Ramachandran plot and check for the presence of amino acid residues in both allowed and banned regions in a descriptive way.

The ligand (SH2) SDF (structure data file format) confirmation was obtained from PubChem (https://pubchem.ncbi.nlm.nih.gov/), and SMILES Translator (https://www2.chemie.uni-erlangen.de/services/translate/) was used to translate it to PDB format. It was then run via the energy reduction procedure of the Avogadro platform (https://avogadro.cc/). For each of these compounds, the MMFF94 force field and the steepest descents were used.

### 2.11 Molecular docking studies

The virtual screening tool Python prescription (PyRx) 0.8 was used to perform molecular docking research using Autodocking vina (22). Positions on the X-, Y-, and Z-axis of the grid were first specified. The centre of the grid was positioned in the active site pocket. In PDBQT format, receptor and ligand files were analysed. The default docking algorithms followed the protocol. Individual docking experiments were performed for each ligand-protein combination (23). The findings were presented by increasing docking energies, The representative value was determined by taking the lowest binding energy of each cluster (24).

### 2.12 Molecular dynamic simulation of *LrSTAT1a* and STAT1a-Src complex system

By using Desmond [D. E. Shaw Research Institute (https://www.deshawresearch.com/resources.html)], Molecular dynamic (MD) simulation of the protein complexes was done. We used the Protein Preparation Wizard to create receptor-ligand complexes. The protein-ligand complexes with the highest scores were simulated using Desmond. A 10 Å box wall distance was used to solvate each protein-ligand combination in an orthorhombic SPC (single point charge) water model. The system was neutralized by adding the correct number of counter ions and a salt concentration of 0.15M. Acetyl and methyl amide were used to cap the STAT1a receptor, while the titratable amino acid residues were left in their dominant condition at pH 7.0. For protein interactions, this system used the OPLS-4 force field. Following the removal of overlapping water molecules, the systems were neutralized using Na+ ions. After the complex system was relaxed with the sharpest downward energy decrease, it was subjected to progressive heating to 310 K while being constrained. The system was simulated with the Berendsen NVT ensemble and kept at 310 K to keep the heavy atoms on the solute. The MDS was conducted under isothermal isobaric ensemble conditions (300 K, 1 atm pressure, and 200 ps thermostat relaxation period). The pressure and temperature scales were regulated using the semi-isotropic Parrinello-Rahman barostat and the Nose-Hoover thermostat for 100 ns at 300 K and 1 atm, respectively. Every 50 picoseconds, step-by-step MD simulation images were captured. Post-MD analysis, which included intermolecular interaction studies, was conducted on the extracted MD trajectories. This analysis encompassed dynamical stability and flexibility.

## 3 Result

### 3.1 *LrSTAT1a* gene comparison with other well-known *STAT1a*

The results of the BLAST analysis suggested the existence of the *LrSTAT1a* gene’s present protein sequence. The *LrSTAT1a* gene had its maximum point of uniqueness with a segment of *Sinocyclocheilus rhinocerous STAT1* (92%) as followed by *Sinocyclocheilus grahami* (90%)>, *Cyprinus carpio* (90%)>, *Carassius auratus* (90%).

### 3.2 *LrSTAT1a* gene identification and expression kinetics from cDNA fragment following Poly I: C induction

**Figure 2:**
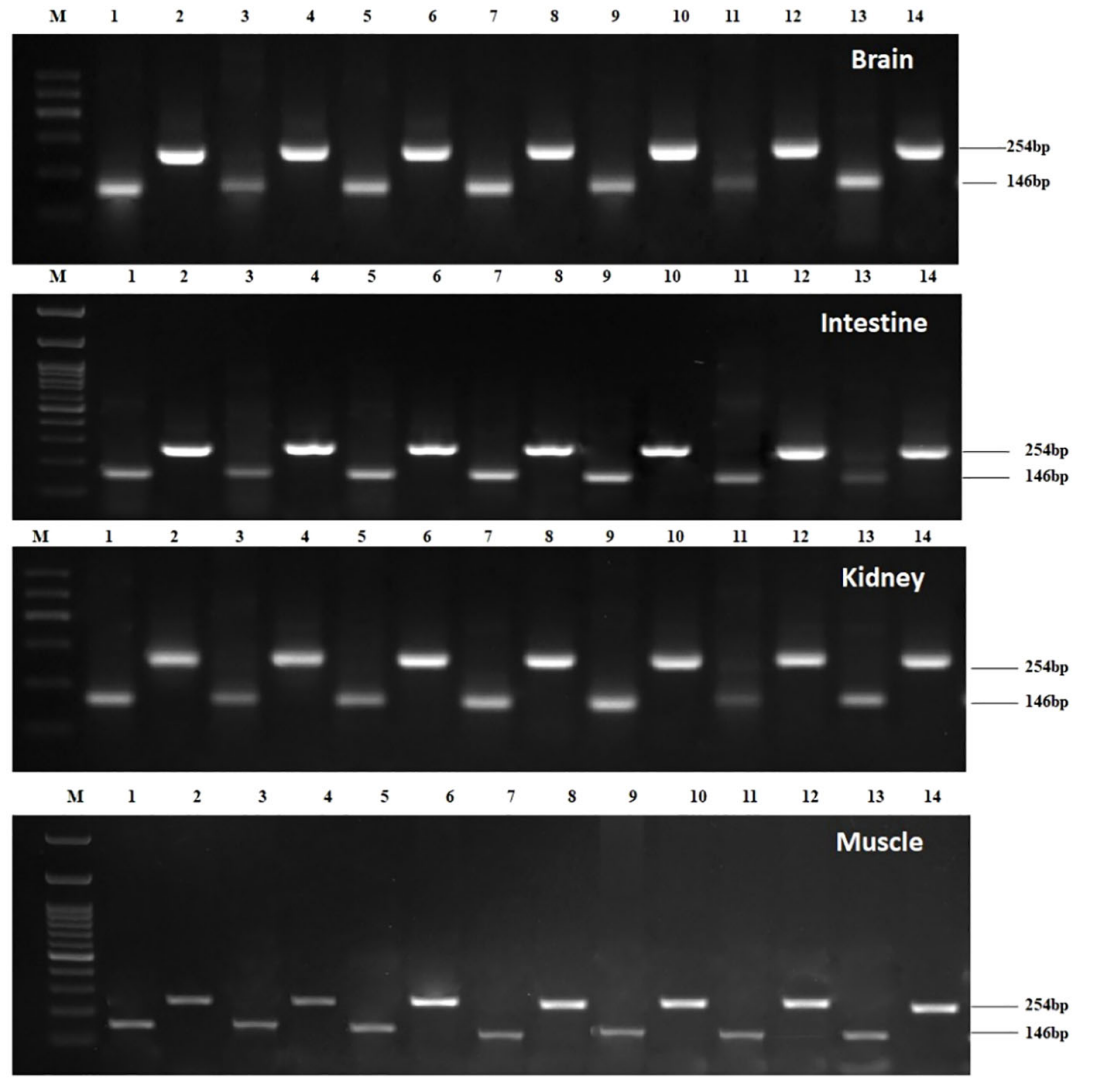
Expression kinetics of Beta-actin and *STAT1a* in different tissues i.e., brain, intestine, kidney, and muscle of *Labeo rohita* followed by semi-qPCR. Brain: Lane M: 100bp Marker; Lane 1: β-actin Day1; Lane 2: *STAT1a* Day1; Lane 3: *β-actin* Day2; Lane 4: *STAT1a* Day2; Lane 5: *β-actin* Day3; Lane 6: *STAT1a* Day3; Lane 7: *β-actin* Day4; Lane 8: *STAT1a* Day4; Lane 9: *β-actin* Day5; Lane 10: *STAT1a* Day5; Lane 11: *β-actin* Day7; Lane 12: *STAT1a* Day7; Lane 13: *β-actin* Day14; Lane 14 *STAT1a* Day14; Intestine: Lane M: 100bp Marker; Lane 1: *β-actin* Day1; Lane 2: *STAT1a* Day1; Lane 3: *β-actin* Day2; Lane 4: *STAT1a* Day2; Lane 5: *β-actin* Day3; Lane 6: *STAT1a* Day3; Lane 7: *β-actin* Day4; Lane 8: *STAT1a* Day4; Lane 9: *β-actin* Day5; Lane 10: *STAT1a* Day5; Lane 11: *β-actin* Day7; Lane 12: *STAT1a* Day7; Lane 13: *β-actin* Day 14; Lane 14 *STAT1a* Day 14; Kidney: Lane M: 100bp Marker; Lane 1: *β-actin* Day1; Lane 2: *STAT1*aDay1; Lane 3: *β- actin* Day2; Lane 4: *STAT1a* Day2; Lane 5: *β-actin* Day3; Lane 6: *STAT1a* Day3; Lane 7: *β-actin* Day4; Lane 8: *STAT1a* Day4; Lane 9: *β-actin* Day5; Lane 10: *STAT1a* Day5; Lane 11: *β-actin* Day7; Lane 12: *STAT1a* Day7; Lane 13: *β-actin* Day 14; Lane 14 *STAT1a* Day14; Muscle: Lane M: 100bp Marker; Lane 1: *β-actin* Day1; Lane 2: *STAT1a* Day1; Lane 3: *β-actin* Day2; Lane 4: *STAT1a* Day2; Lane 5: *β-actin* Day3; Lane 6: *STAT1a* Day3; Lane 7: *β-actin* Day4; Lane 8: *STAT1a* Day4; Lane 9: *β-actin* Day5; Lane 10: *STAT1a* Day5; Lane 11: *β-actin* Day7; Lane 12: *STAT1a* Day7; Lane 13: *β-actin* Day14; Lane 14 *STAT1a* Day14.

Constitutive expression of *STAT1a* was deliberated in the brain, spleen, liver, intestine, heart, kidney, gill, and muscle of *L. rohita* for fourteen days post-induction. By using the semi-quantitative PCR approach, it was possible to determine that the brain, muscle, kidney, and intestine had the highest levels of *STAT1a* expression on day two after Poly I: C induction (**Figure 2**), which was confirmed in qPCR. The expression decreased after third day. While the mean *STAT1a: β-actin* ratio in the treated groups was after Poly I: C injection, the mean ratio on the second day was recorded to be approximately 12.19 and decreased to 1.40 between day 5 and day 14. Many of the untreated control groups showed no *LrSTAT1a* mRNA expression. However, based on mRNA configuration, statistical analysis showed a positive difference (p<0.05) between fish injected with poly I: C and control fish starting on day two and continuing until day four.

### 3.3 *LrSTAT1* expression and qPCR following Poly I: C induction

**Figure 3:**
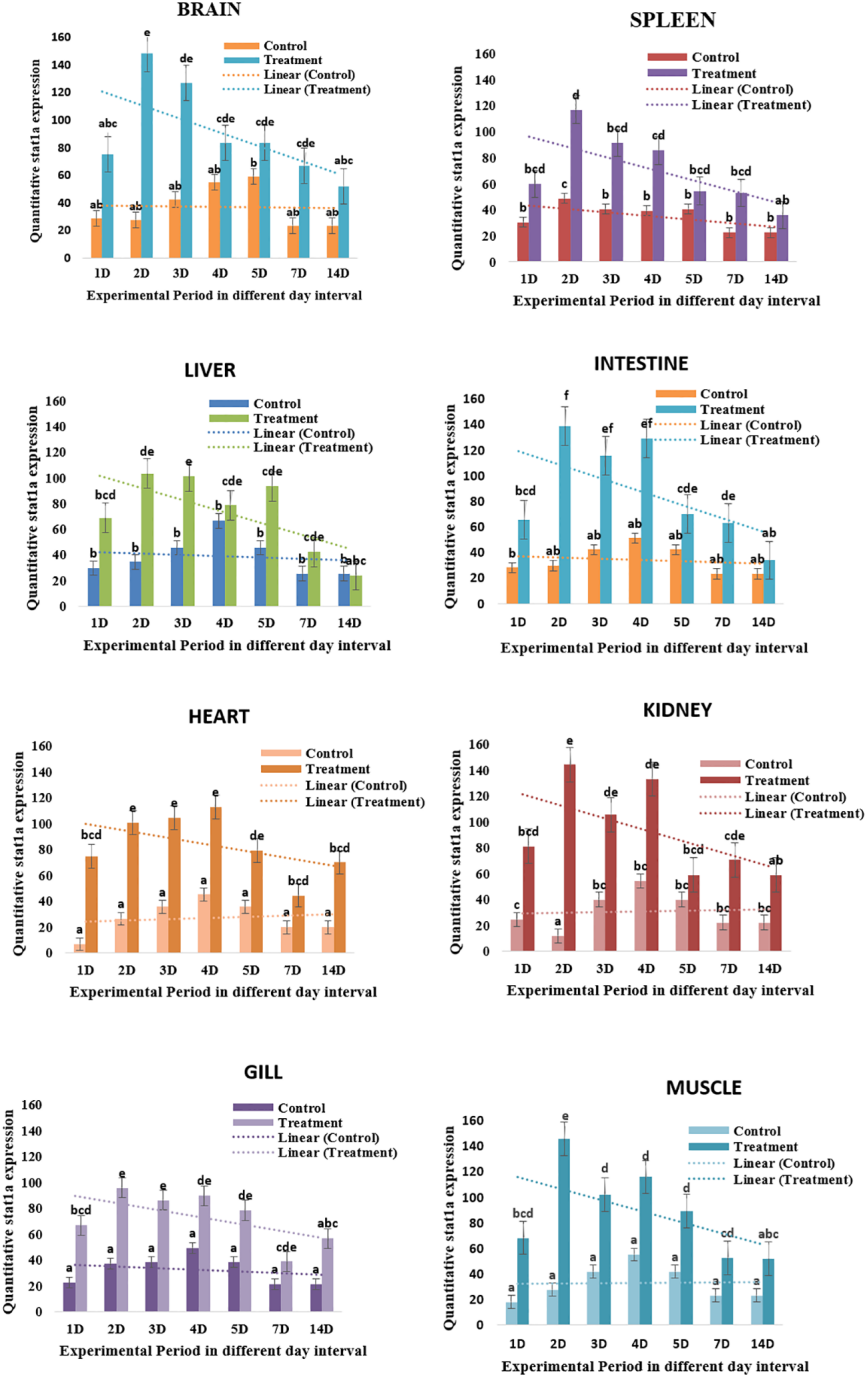
Quatitative *LrSTAT1a gene* expression in different tissue. Changes in *STAT1a* expression over a 14-day experiment in treated and control *L. catla* tissues (Brain, spleen, liver, intestine, heart, kidney, gill, and muscle). Lowercase letters, mean the statistical analysis.

The expression of the *LrSTAT1a* gene in the brain tissues on day 2 (6.1fold) and then rapidly decreased on day 7 (2.7-fold change at lowest) and was nearly undetectable on day 14. *LrSTAT1a* expression exhibited a decreasing pattern on the spleen. The expression of *LrSTAT1a* in the liver tissues followed a similar trend. On day 2 (2) day 3 (1.9), and the heart tissue expression pattern peaked up to day 4, then it gradually declined until day 14 (**Figure 3**).

### 3.4 *STAT1a* gene, expression dynamics in different tissue of *L. rohita*

**Figure 4:**
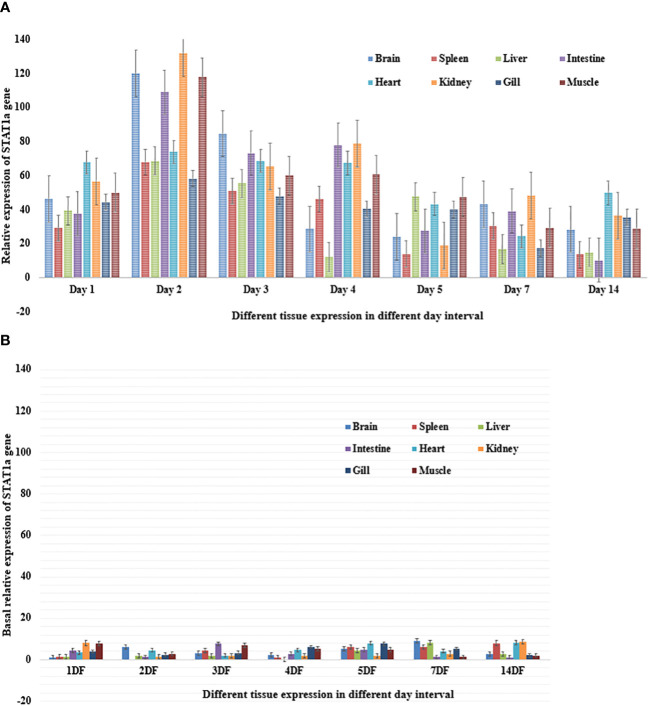
**(A)** Relative expression of *STAT1a* gene in different tissue of *L. rohita* after induction of poly I: C over an experimental period of fourteen days. The information is displayed as mean relative expression ± (n=5) for the brain, spleen, liver, intestine, heart, kidney, gills and muscle.The standard error bar is based on real time values and being represented as mean ± standard error. **(B)** Basal relative expression of *STAT1a* gene in different tissue of *L. rohita* over an experimental period of fourteen days. The information is displayed as mean relative expression ± (n=5) for the brain, spleen, liver, intestine, heart, kidney, gills and muscle. The standard error bar is based on real time values and being represented as mean ± standard error.

Poly I: C encouraged *STAT1a* to express itself steadily and well; it peaked on day 2 and returned to normal on day 14. A semi-quantitative PCR was performed after poly I: C induction to determine the intensity of *STAT1a* expression in each of the vital organs (brain, spleen, liver, intestine, heart, kidney, gill, and muscle). The development of a quantitative real-time PCR is underway to assess the level of *STAT1a* expression in the key organs after Poly I: C induction. For the duration of the investigation, fish induced by poly I: C were taken into account, and fish injected with phosphate buffer saline (PBS) served as the baseline treatment control group. The results of the semi-quantitative real-time PCR reaction matched the expression of *STAT1a* transcript analysis by RT-PCR across all tissues. Fish that were given PBS as a control showed less *STAT1a* expression. On day 2, *STAT1a* expression was remarkably prevalent throughout all tissues, but maximal up-regulation of STAT1a expression was observed in the kidney, brain, and muscle, whereas the spleen, liver, heart, and gill also showed the greatest *STAT1a* expression, respectively (**Figure 4**).

### 3.5 RACE-based *STAT1a* transcript characterization

**Figure 5:**
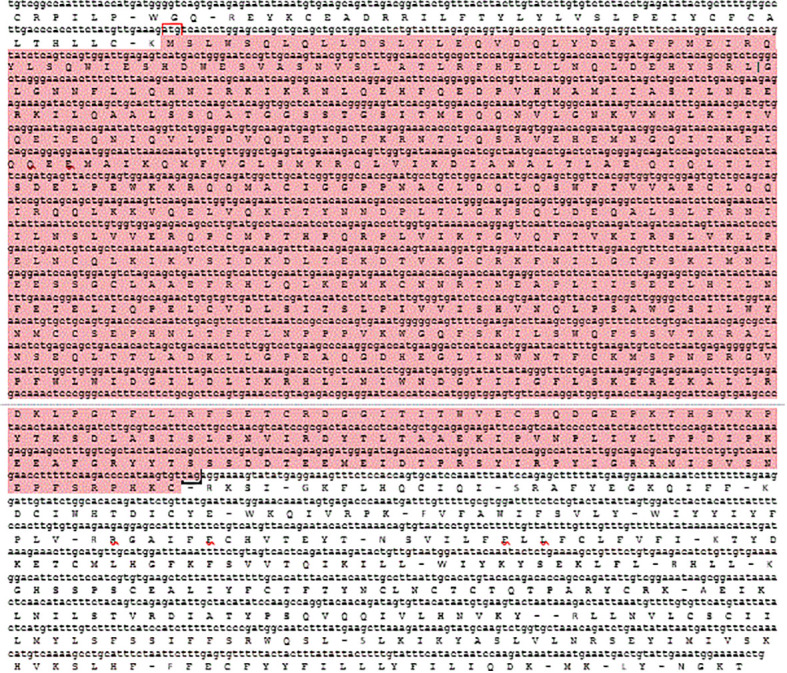
The cDNA nucleotide and predicted amino acid sequence of *LrSTAT1a*. The ORFs are shown in highlighted. Red box indicate the translation initiation codon (ATG) and black box indicated stop codon (TAG) sysmbole indicating. Untranslated and coding regions are in lower and upper case, respectively.

**Figure 6:**
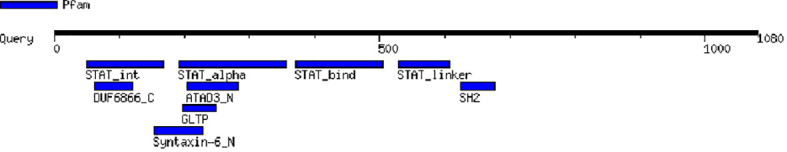
Different motif regions of STAT1a protein predicted by motif tool of Genome Net.

**Figure 7:**
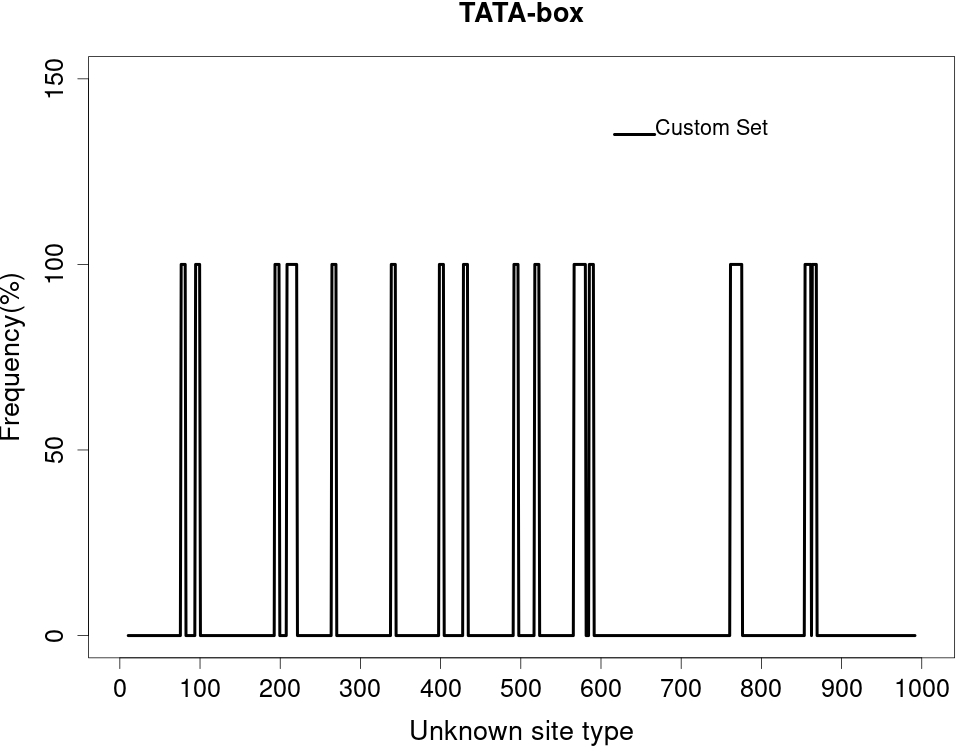
Several TATA box present in predicted promoter region of STAT1a gene.

**Figure 8:**
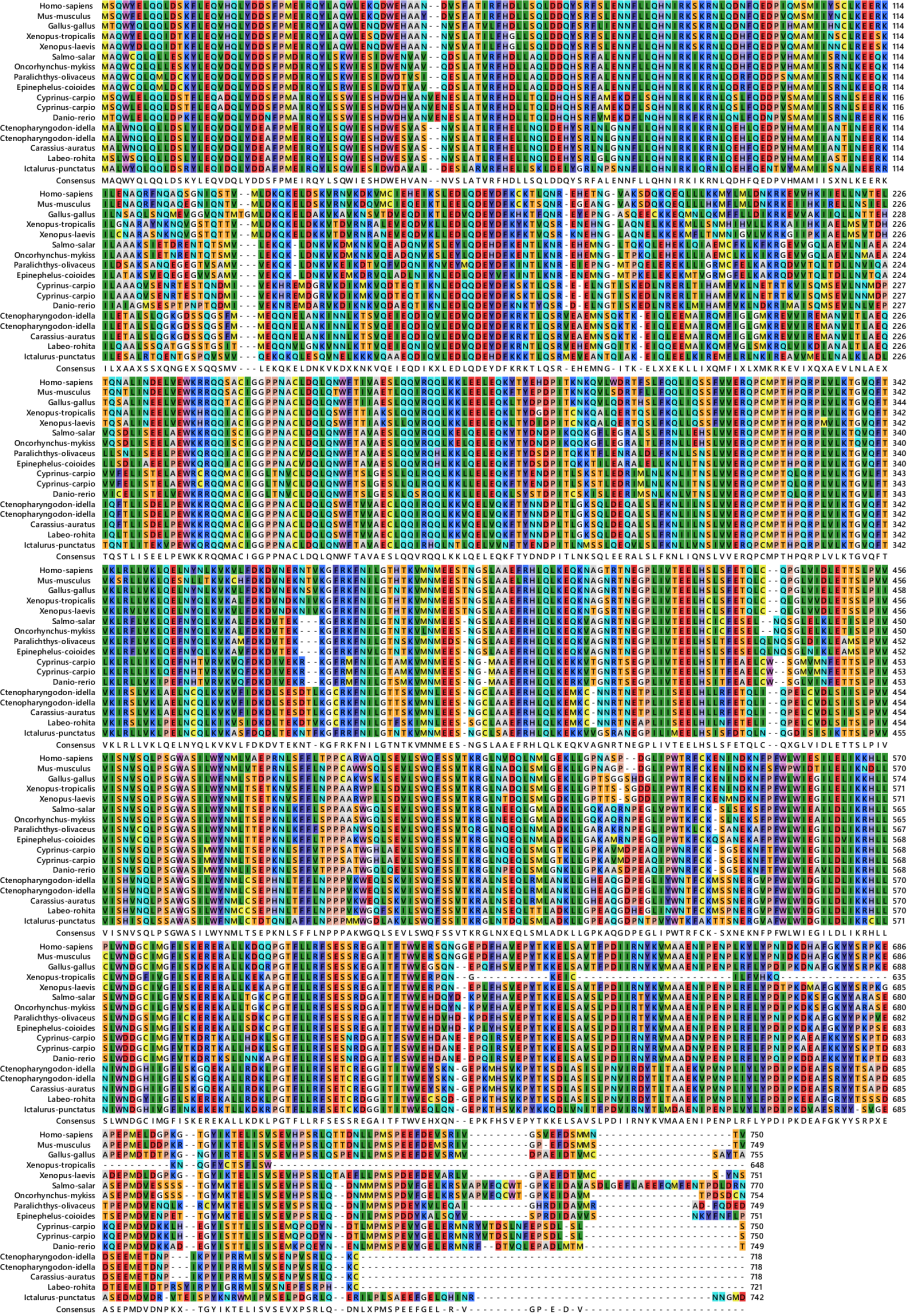
Multiple alignment of STAT1a amino acid sequences from different species (Homo-sapiens, fishes and birds).

After the unknown ends of the cDNA were cloned using RACE and sequencing of rapid amplification of the cDNA ends result, the entire sequence of 3238 bp was acquired (**Figure 5**). The molecular weight (MW) and isoelectric point (pi) of STAT1a protein calculated by the Expasy tool were 123144.46 Da and 8.32, respectively. The grand average of hydropathicity (GRAVY) value of STAT1a protein calculated by ProtParam was -0.101. Nine motifs were found in the STAT1a protein (**Figure 6**). There were several TATA boxes (length 15) found in the predicted promoter site of *STAT1a* gene (**Figure 7**). This was submitted to the NCBI GenBank with Accession Number OQ868190. An overlapping PCR fragment was spliced and combined to determine the whole sequence. Through ATG as starting codon at 145 base pairs and TGA as a termination signal at 2310 base pairs, an untranslated 5’ primary region of 42 base pairs and an untranslated 3’ region with 318 base pairs, *STAT1a* transcript exhibited 144 to 5’-UTR, an ORF possessing 2166 base pairs, the encoding with 721 amino acids protein of 928 base pairs, to 3’-UTR, which contains stop signals and a characteristic poly-adenylation signalling location (AATAAA) and In the **Figure 8** showing multiple alignment of STAT1a amino acid sequence from different species (i.e., Homo-sapiens, fishes and birds).

### 3.6 Theoretical modelling of *LrSTAT1a* protein

**Figure 9:**
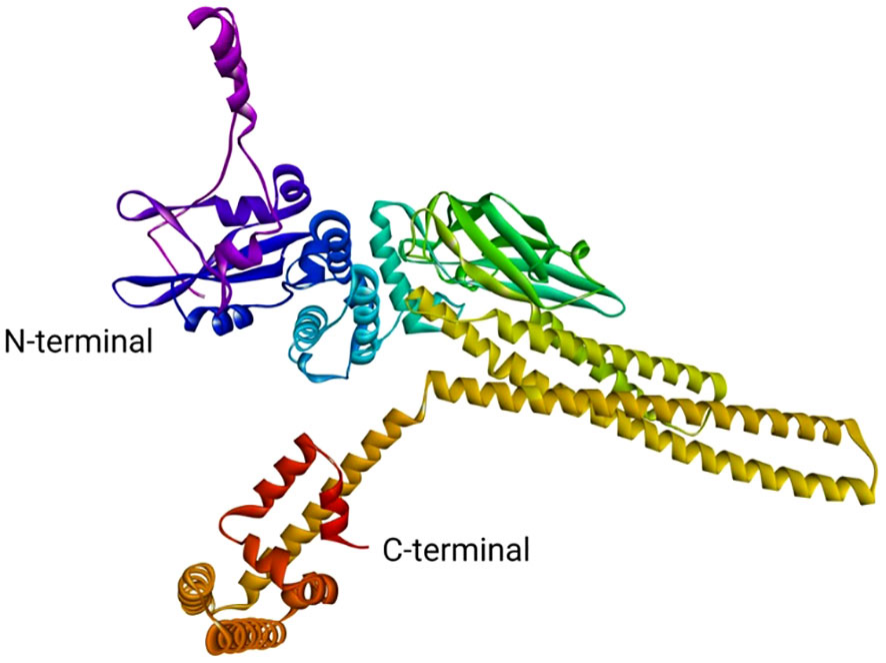
Theoretical model of STAT1a.

The STAT1a protein is a member of the STAT family of proteins, which are transcription factors that play important roles in signal transduction and cellular communication. STAT1a is activated by phosphorylation by Janus kinases (JAKs) in response to various cytokines and growth factors. Once activated, STAT1a dimerizes and translocated to the nucleus, where it binds to specific DNA sequences to regulate gene expression. The 3D structure of the STAT1a protein (**Figure 9**) is typically shown as a ribbon diagram, where each amino acid residue is represented by a coloured ribbon. The structure can be divided into several domains. The N-terminal domain typically contains the DNA binding site and is often located at the N-terminus (beginning) of the protein. In the ribbon diagram, it would likely be represented by a distinct section, potentially with a different colour than the neighbouring domains.SH2 domain: This domain is responsible for protein-protein interactions and is involved in the dimerization of STAT1a molecules. It is usually located near the N-terminus but not at the very beginning. Look for a compact, folded region in the ribbon structure. WHSH domain: This domain is also involved in protein-protein interactions and is thought to play a role in the regulation of STAT1a activity. It is often located between the SH2 domain and the C-terminal domain. C-terminal domain: This domain contains the phosphorylation site for JAKs and is responsible for the protein’s activation. It is typically located at the C-terminus (end) of the protein and might be another distinct section with a different colour in the ribbon diagram. It is important to note that the specific location and appearance of these domains can vary depending on the protein and the way it is visualized.

The Ramachandran plot is a valuable tool for protein structure analysis and validation. It can be used to identify errors in protein models and to assess the quality of protein structures. The allowed regions of the Ramachandran plot are shown in the image as the green and yellow shaded areas. These regions represent the combinations of φ and ψ angles that are sterically allowed, meaning that there are no steric clashes between atoms in the protein. The disallowed regions, which are not shaded, represent combinations of angles that are not possible due to steric clashes.

**Figure 10:**
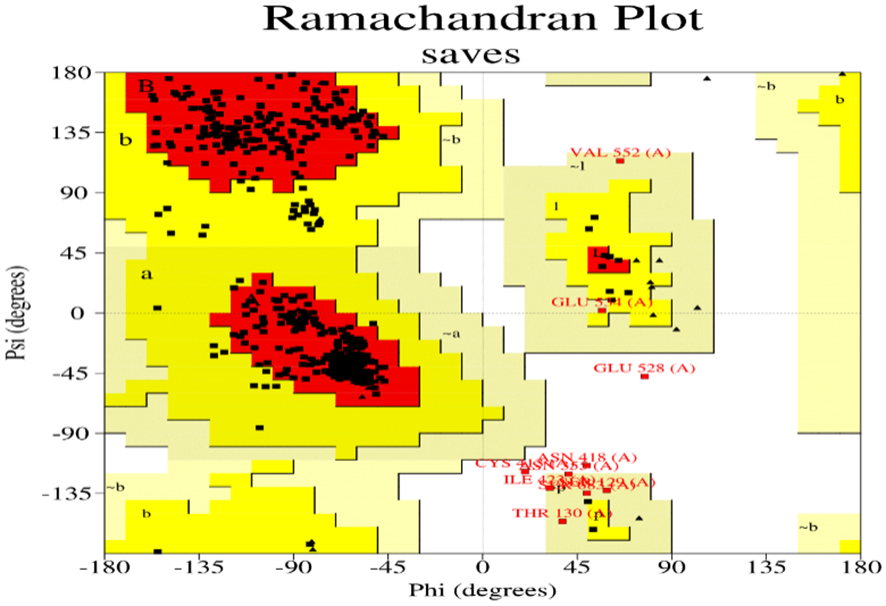
Ramachandran plot of signal transducer and activator of transcription 1a.

The Ramachandran plot (**Figure 10**) of the model indicated that 91.8% of the residues were located in the core region, 6.6% in the allowed regions, 1.1% in the generously allowed regions, and 0.5% Residues in the disallowed region. Thus, the stereo chemically unstable areas contain ten residues. Thus, additional energy minimization and refinement were applied to these residues. 91.8% of the residues were discovered to be located in the core region after the final model was analysed. The overall shape of the projected structure was stabilized and optimized by the displacement of residues from the prohibited zone to the allowed region.

### 3.7 Molecular docking analysis

**Figure 11:**
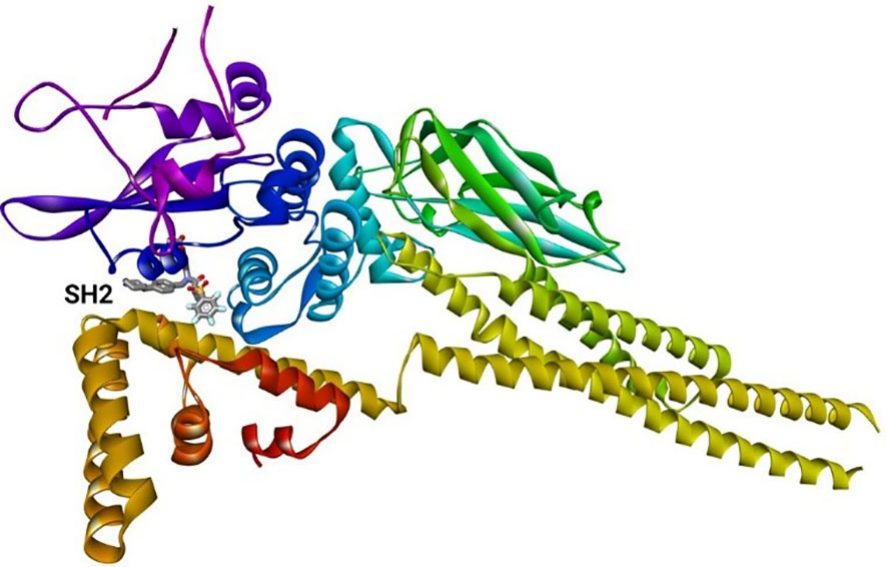
3D crystal structure of signal transducer protein with their respective binding site.

**Figure 12:**
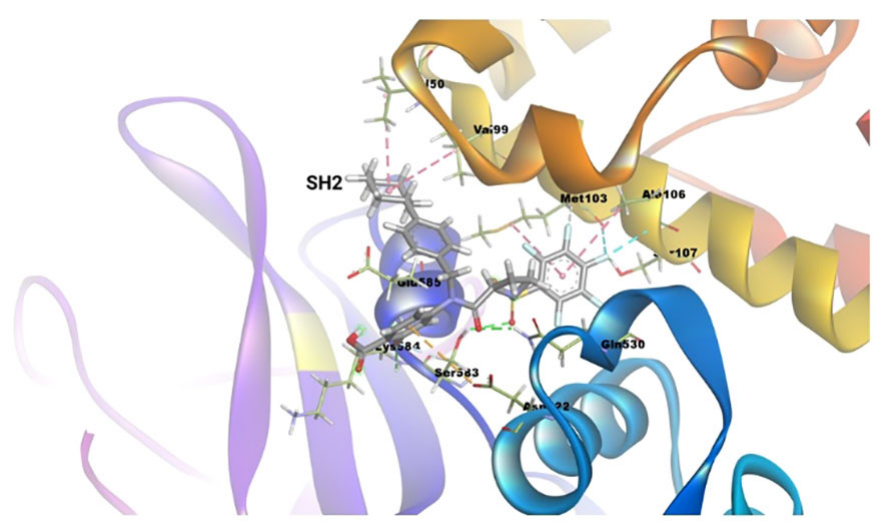
3D interaction diagram of the docked complex (STAT1a SH2 Domain).

**Figure 13:**
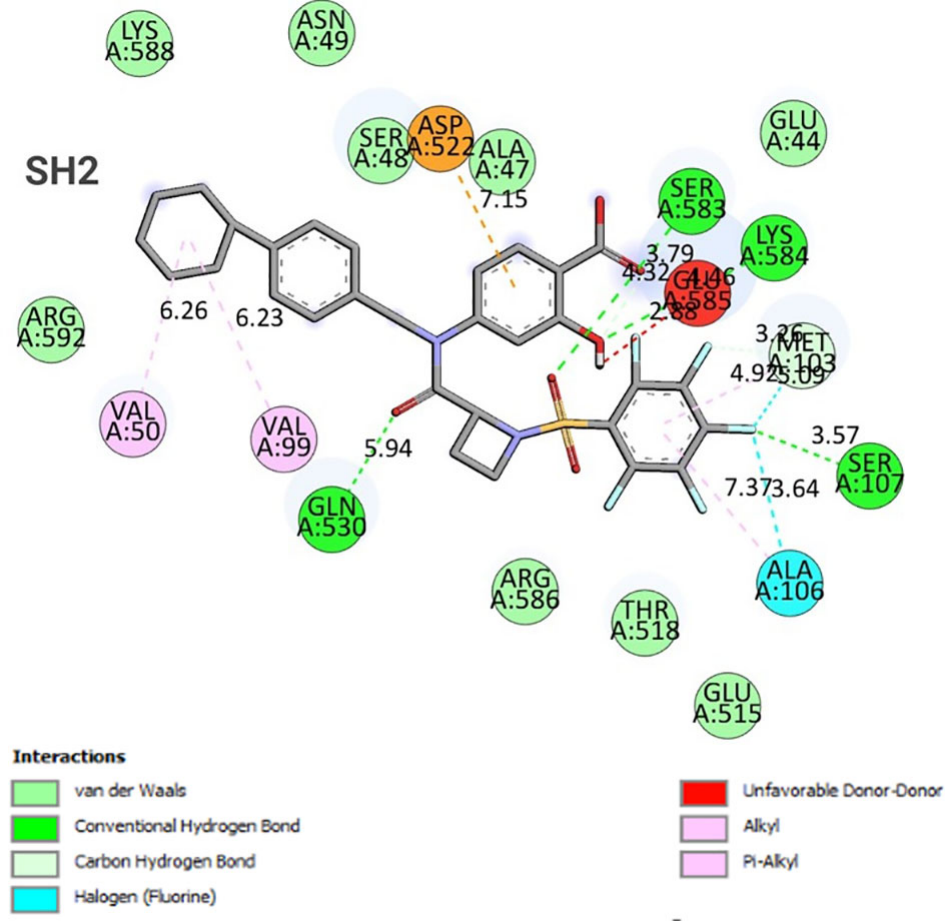
2D interaction diagram of the docked complex (STAT1a - SH2 Domain).

The binding energy between SH2 and STAT1a was -9.7 Kcal/mol. The amino acid residues SER107, GLN530, SER583, LYS584, MET103, and ALA106 form a typical H-bonding with bond lengths of 2.36 Å, 2.17 Å, 2.57 Å, 2.41 Å, 2.19 Å, and 2.47 Å, in that order. Hydrophobic interaction involved residues VAL50, VAL99, MET103, and ALA106 with bond lengths of 5.40 Å, 5.17 Å, 4.78 Å, and 5.40 Å, respectively. Two Halogen (Fluorine) interactions MET103 and ALA106 with bond lengths of 3.37 Å and 3.62 Å, and an Electrostatic interaction (ASP522) with a bond length of 4.98 Å were found by docked complex analysis. **Figure 11** displays the binding location of STAT1a proteins, whereas **Figures 12**, **13** depict the docked complex’s 3D and 2D interactions (STAT1a SH2 Domain).

### 3.8 Molecular dynamics simulations

**Figure 14:**
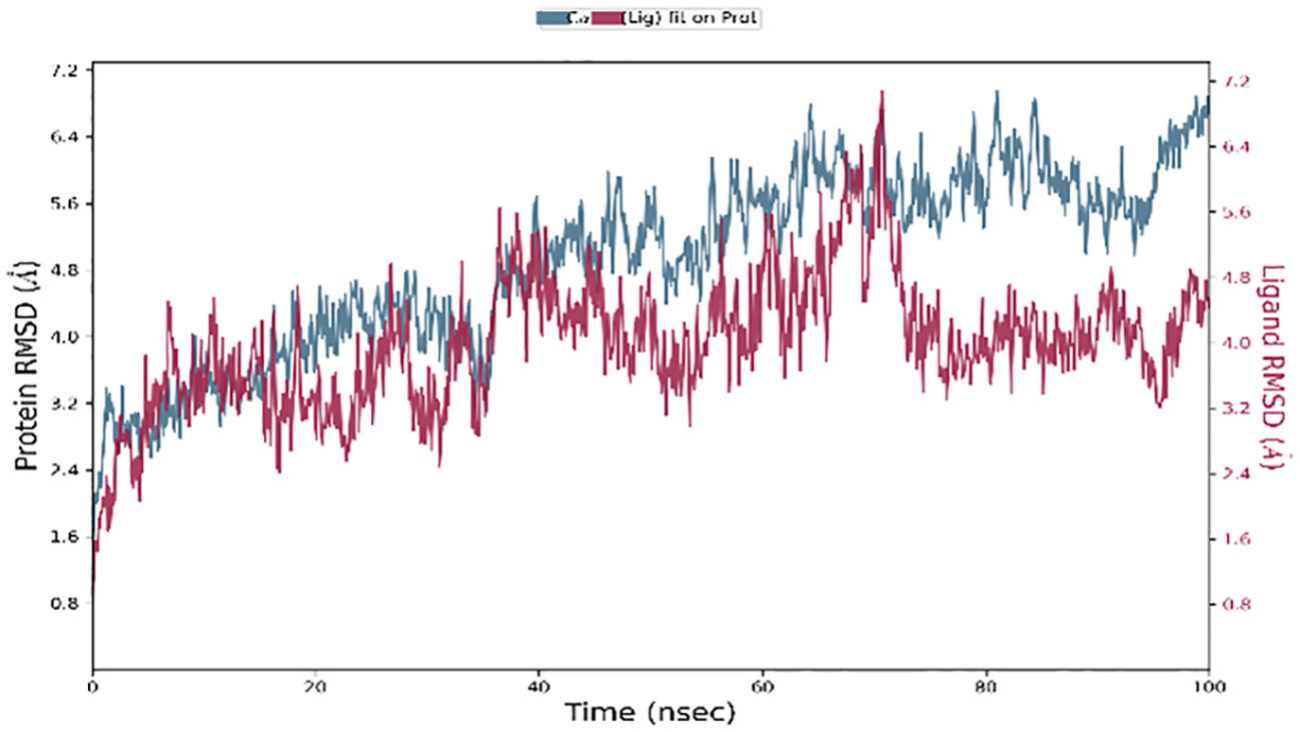
RMSD plot of backbone atoms with respect to time water of STAT1a protein.

While molecular docking is a quick and efficient method for determining the binding position of a ligand with the active site of a protein, it does not capture the conformational changes that occur during ligand-protein interactions. A molecular dynamics (MD) simulation was performed to evaluate conformational changes more accurately (25). One hundred nanosecond molecular dynamic simulations were executed to evaluate the flexibility, stability, and system stability of the bound ligand SH2. The molecular dynamic stability of each complex was investigated by computing the backbone root mean square deviation (RMSD) of the protein relative to its initial conformation. All systems reached equilibrium after 60 nanoseconds of simulation relative to the reference frame at time 0 nanoseconds, as shown in **Figure 14**.

The left Y-axis of **Figure 14** showed the protein RMSD, whereas the right Y-axis showed the ligand RMSD profile aligned on the protein backbone. All frames from the 100 ns trajectory are aligned on the reference frame backbone. Within the initial two nanoseconds of the trajectory, the variation in ligand RMSD was observed. Nevertheless, the modifications centered around 0.15 Å, suggesting that the complex had not experienced substantial alterations to its structure. In addition, fluctuations in ligand RMSD on the order of 4.5 Å that persist for a duration of 40 ns are initiated by the simulation at 5 ns. Until the conclusion of the 100-ns simulation, the ligand RMSD remained constant after 40 ns. Upon concluding the investigation, a slight divergence of 70 nanoseconds was identified. The fluctuation is considered insignificant since it falls within the permissible range of 1 to 10 Å.

**Figure 15:**
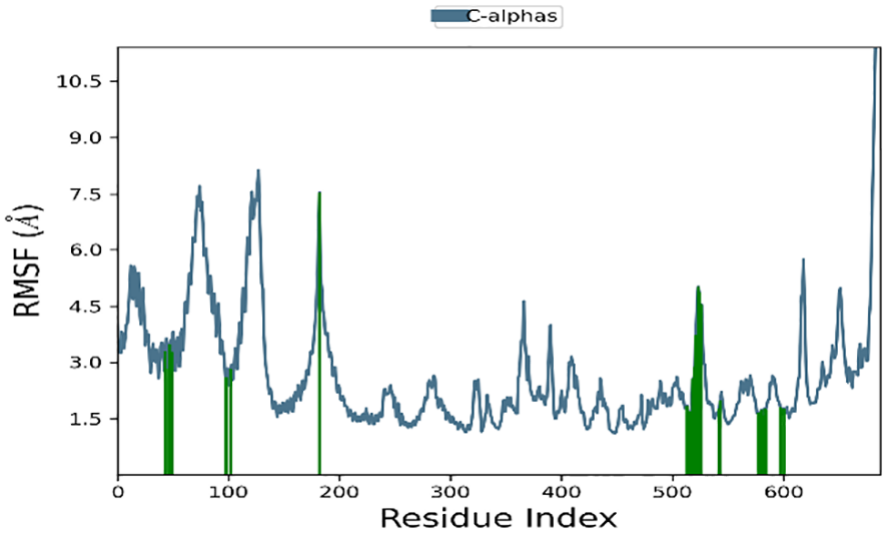
RMSF plot of the backbone of STAT1a protein.

**Figure 16:**
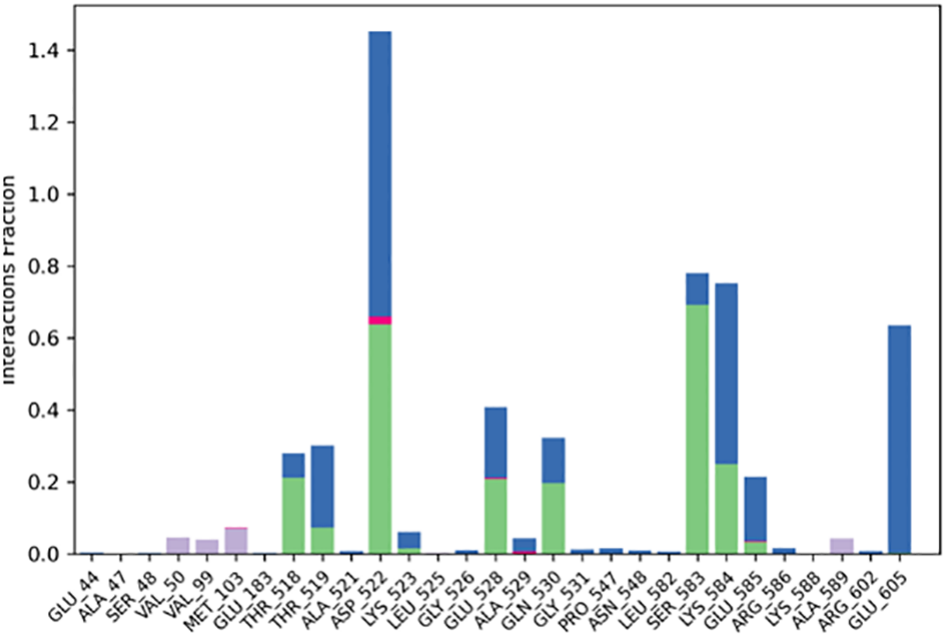
Protein-ligand interaction histogram obtained from MD Simulation of STAT1a with SH2.

**Figure 17:**
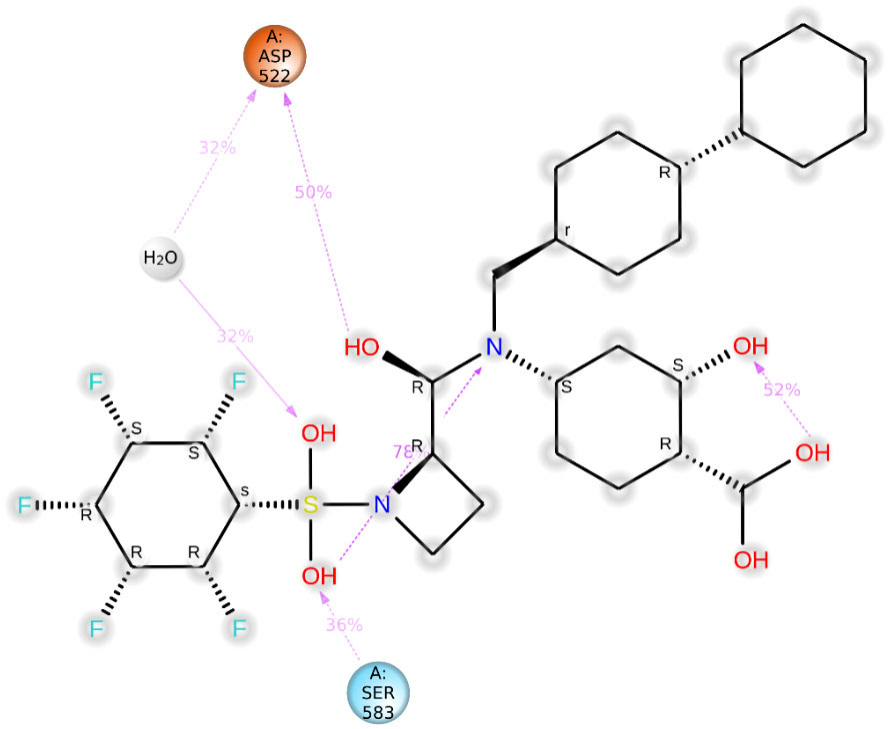
Detailed schematic interaction of STAT1a protein with SH2.

Significant local changes in the RMSF (**Figure 15**) pattern of C-atoms were observed in the STAT1a receptor systems following phytocompound binding, as compared to the other receptor systems. At the N-terminal end of each system, the amino acid residues implicated in interaction with the compounds exhibited significant changes (RMSF increases), indicating their involvement in ligand recognition. Amino acids varied between 1.5 Å and 10.5 Å in the RMSF of the STAT1a receptor system, but it steadied and remained consistent between 1.5 Å to 6.0 Å **Figure 15**. Residues of the binding site of the docked protein and ligand are illustrated in the schematic interaction diagram in **Figures 16**, **17**. The stability of the ligand-protein complex is shown to be affected by interactions involving hydrogen, hydrophobic groups, and ions. Ligand binding requires the formation of H-bonds. Hydrogen-bonding properties greatly influence drug selectivity, metabolization, and adsorption, making them crucial for drug design. The STAT1a complex interactions are shown in **Figure 16** as normalized stacked bar charts over a 100 ns trajectory. Water bridges (TH518, THR519, ALA521, ASP522, LYS523, GLU526, GLU528, ALN529, GLN530, GLY531, PRO547, ASN548, LEU582, SER583, LYS584, GLU585, ARG586, ARG602, GLU605), ionic bond interactions (ASP522, GLU528, ALA529), hydrogen bonds (THR518, THR519, ASP522, LYS523, GLU528, GLN530, SER583, LYS584, GLU585), and hydrophobic interactions (VAL50, VAL99, MET103) are the many forms of these connections. Hydrogen bonds, hydrophobic contacts, and water bridges were the most prominent during the MD simulation, as seen in the bar chart. **Figure 17** shows the interaction with the stat1a complex, namely molecular interactions with residues ASP522, SER583, which were constant during the 100 ns simulation.

## 4 Discussion

This is the first report to document successful cloning and identify the whole cDNA *of L. rohita’s STAT1a*. The known STAT1 sequences from other animals were compared with the amino acid sequence of LrSTAT1a. The fact that the sequences of *LrSTAT1a* and other *STAT1* were so similar suggests that the cloned gene used in this investigation is authentic *STAT1a* from *L. rohita.*


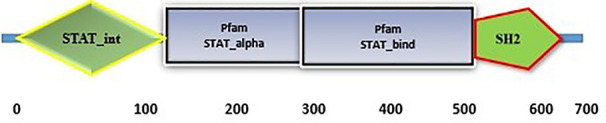


*LrSTAT1a*’s domain structure shares a great deal of similarities with other STATs. *LrSTAT1a* has four key domains in the amino acid sequence. SH2 domain and DNA binding domain. The NH2 terminal contains the protein interaction domain, which enables dimers to bond with one another (26). The coiled-coil domain and the all-alpha domain are interchangeable. Particular interactions between this domain and other proteins (27). The transcription of genes depends on the DNA binding domain (28). In both homo- or heterodimerization and STAT signalling, the SH2 domain is crucial similar (29, 30). These features suggest that communication from receptor phosphorylation to DNA transcription was mediated by *LrSTAT1a*.

Innate immunity and IFN function depend on STAT1a. However, few studies have examined the expression of the *STAT1a* gene in various fish organs. In this work, we used qPCR to investigate the expression of *STAT1a* mRNA in several *L. rohita* organs. As demonstrated in **Figure 4**, STAT1a is highly expressed in every tissue. Natural killer cells and plasmacytoid dendritic cells, which are the major sources of interferons, are abundant in the liver (31). In addition, we observed that the expression of *LrSTAT1a* mRNA was similarly high in the brain and muscles. Since muscles are the first line of defence for fish against harmful microorganisms against harmful microorganisms for fish, high levels of *LrSTAT1a* are necessary for quick and effective innate responses in muscles. In a similar vein, one immunological organ that is crucial to fish’s adaptive immune response is the kidney. Consequently, the high expression of *LrSTAT1* is not surprising (32). These findings align with findings from studies on mandarin fish and olive flounder (16, 17). Thus, our findings suggest that tissue defence against pathogens depends on high levels of *LrSTAT1a* expression.

According to the earlier research, some *STAT1* are engaged in the early phases of embryonic development (33). In invertebrates, such as brine shrimp and fruit flies, the *STAT* gene controls the development of the embryo and the immune system (34–37). For vertebrates like mice, this is not the case. Mice lacking *STAT1* exhibited normal birth rates and no obvious developmental abnormalities, but they developed severe issues with their IFN-dependent immunological responses to viral infections (8). Furthermore, a subsequent investigation discovered that animals with *STAT3* loss exhibit early embryonic lethality (38). These findings suggest that each form of *STAT* may have a distinct physiological role in mammals. For instance, *in situ*, hybridization tests reveal that *STAT3* is substantially expressed during zebrafish embryogenesis before 6 days post fertilisation (dpf), but not *STAT1* (4). Olive flounder has yielded comparable outcomes. Although *STAT1* expression was seen in all developmental stages in these publications, there wasn’t enough data to make inferences from the fertilised egg to the larvae stage (17) It was possible to detect *LrSTAT1* expression in every embryonic stage, including the fertilised egg. The high level of *LrSTAT1* expression at 120 hpf suggested that *LrSTAT1* would become increasingly significant after embryogenesis. A prior work stated that *STAT1* is necessary for dendritic cell maturation (39). Thus, we propose that *LrSTAT1a* is likewise critical for the development of immunity in *L. rohita.*

In this study, *LrSTAT1a* expression was considerably induced by poly I: C stimulation of *L. rohita*. By interacting with pattern recognition receptors (PRR) like TLR3, Melanoma Differentiation Associated Protein-5 (Mda5), and Retinoic Acid Inducible Gene I (RIG-I), which recognise double-stranded RNA, the adaptor protein T1CAM-TRIF is recruited to activate interferon regulatory factor (IRF) 3, which induces interferon production (40, 41). Poly I: C is an artificial double-stranded RNA and simulated RNA virus infection (42). Through the JAK-STAT signal pathway, IFN suppresses viral infection by causing the expression of downstream genes like Mx. Salmon, turbot, rainbow trout, yellow croaker cod, and rainbow trout have all been shown to use this signalling pathway (43–48), Since poly I: C has a strong antiviral effect, treatment may also be used to protect against getting a virus (49). Our tests show that poly I:C is the most effective, which might be a bit overstated *LrSTAT1a* expression.

## 5 Conclusion

The molecular characterization clearly shows of *LrSTAT1a* expression after induction of Poly I: C. A combination of biotechnology and bioinformatics is used to describe the molecular mechanism of *LrSTAT1a* protein-mediated immunological protection, highlighting the dynamics and development of Src homologous domain linkage. After the cDNA of the LrSTAT1a protein was cloned, expression was observed in various tissues. To identify the close structural homologs among mammals, a comparative analysis based on sequence-structure analysis was conducted. The generated protein model revealed that the modelled *L. rohita* STAT1a is a member of a sizable SH2 family. The modular organization of the *L. rohita* STAT1a protein is explained by three functional domains identified in this study, among which the SH2 binding pocket is crucial for antiviral activity. Using the in-silico analysis methods, Src activity-ending helps to ensure that significant functional residues are involved. Additionally, following the 14-day induction of Poly I: C in many tissues, including the brain, liver, muscle, spleen, heart, gills, and kidney, the study demonstrated tissue-specific expression of *STAT1a*.

## Data availability statement

The original contributions presented in the study are publicly available. This data can be found here: NCBI GenBank under the accession number OQ868190.

## Ethics statement

The animal study was approved by ICAR-Central Inland Fisheries Research Institute. The study was conducted in accordance with the local legislation and institutional requirements.

## Author contributions

BD: Conceptualization, Funding acquisition, Supervision, Visualization, Writing – review & editing. SPa: Data curation, Formal analysis, Investigation, Methodology, Software, Validation, Writing – original draft, Writing – review & editing. SPr: Formal analysis, Methodology, Software, Writing – original draft. SR: Formal analysis, Methodology, Software, Writing – original draft. MK: Methodology, Writing – review & editing. DM: Methodology, Writing – review & editing.
